# Supplementary material for: Patterns of Intron Gain and Loss in Fungi
Source: PLoS Biol. 2004 Nov 30;2(12):e422. doi: 10.1371/journal.pbio.0020422 (PMC532390; doi:10.1371/journal.pbio.0020422)
Supplement: Table S1 — Also available at http://genes.mit.edu/NielsenEtAl/. (4.3 MB ZIP). [file pbio.0020422.st001.zip › NielsenEtAl/html/710.html]

AN1167.1.NCU02634.1.MG04443.1.FG01009.1


```
 CLUSTAL W (1.82) Multiple Sequence Alignments - Introns Inserted


Sequence 1: MG04443.1	380 aa
Sequence 2: FG01009.1	389 aa
Sequence 3: NCU02634.1	393 aa
Sequence 4: AN1167.1	382 aa
Alignment Length: 406 aa
Number Identitical Residues: 145 aa
Alignment Score (without introns) 7799


MG04443.1 	MSINPLITFKAGR2----~---KPAKVKCLPEPGYIYLYSDDG1LINFCWRPRSQAIDDE
NCU02634.1	MPISPIITFKAGI~CDVD0QTSKPYKVTPQARPGYIYLYSDDD1FIHFCWRPRDAPADE-
FG01009.1 	MPISPILTFKAGQ~CEVD0TSSKPYKVKPQSEPGYIYLYSEDD1LVHFCWRKRTEPLDN-
AN1167.1  	MSITPIITFKAGI~CDLE0TQSSGVHAKPQPTPGYLYLYSEDE~LIHFCWRPRSAPHTE-
          	*.*.*::*****  .. .   :.  :..  . ***:****:*  :::**** *  .  : 

MG04443.1 	PPLSLVMVPGDGRFVPYQ--PDDQPSGKTNGRILVLKFLSSSTRHLFWMQSKPQSTSGDP
NCU02634.1	PELDLVMIPEDGHFLPYDTKSPAQASAKTNGRIFSLKFSSSSQRHIFWLQSKPQSPNGDP
FG01009.1 	PELDLIMVPTDGSFTPYESTTSSEPTSKTDGRIFVLKFSSSSQRHIFWLQSKPQSENGDA
AN1167.1  	PELDLVMVPSDATFTPYR--ADSKP---TNGRIYVLKFSSSSQRYLFWLQSKSQHENADP
          	* *.*:*:* *. * **   .  :.   *:***  *** *** *::**:***.*  ..*.

MG04443.1 	AWFSPRDRKITDLVDQLLSGEEVDVQQELAEARSSN-CRDDEDDDDETMEDADGQGRSGQ
NCU02634.1	SFLSPRDLKIGEIVDKLLQGYEVDVNRELASLSSGGGNNRRDDDDDETMEDVEGHGDPNA
FG01009.1 	AYYSPRDRKIGDIVNRLLQGEEVEVAEEMATVRND----DQDDDEDEAMEDADNQR---P
AN1167.1  	AFWSARDLKLGWIVNKLLQGEEFDVEREIATIPKGP----SNGDDDETMEDVEGVDHDPN
          	:: *.** *:  :*::**.* *.:* .*:*   ..      :.*:**:***.:.      

MG04443.1 	RHQSASGG-AGADATGGDVRREGEDSREGGADGARA2RGG---ATDAATIVQNLLDSLAG
NCU02634.1	HHEGGTGG-AGPDATGGDVRREGEDAREGGADGARA2ANATNAEADAAAVVRNFLESLKG
FG01009.1 	REQRGSGG-AGPDATGGDVREEGEGSREGGADGARA2VSSSAPGTDASAAVRSFLDSLRG
AN1167.1  	HPHGGSGGGAGPDATGGDVREEGQESREGGADGGRA2AAV---DSDASAVVQNFLRSFGG
          	: . .:**.**.********.**: :*******.**        :**:: *:.:* *: *

MG04443.1 	SSGGGNQQQQSQAQGKVYPLLSDLLETPTTIPVIDGADETYIDNLLDLLPATIVVMAAD-
NCU02634.1	TQGRAGG--QAQAQGKSYPLLNDLLESSVTIPMLDSATDEYVDNLLNYLPPTVIVMSQQG
FG01009.1 	QSGISGGQQQQQSADKAYPYLNHLLPTSITVPMIDSAPEEFADTLISFLPPAVIVLASGS
AN1167.1  	N--------QSQQAEQPFTTLQDLLSPSTTLPFLDTLNVEGVDNLLKFLPQELLILALQM
          	         * *   : :. *..** .. *:*.:*       *.*:. **  :::::   

MG04443.1 	-DEFDG-EANPPAA--AAAKASMSLPEKKALLKQVLRSPQFHQSLSSLSMALRDGGLPTI
NCU02634.1	ASGTDAIEVEPSAASVEAARQAMSQDQKRALLKKVLRSPQFTQSLASLTIALRDGGLPNI
FG01009.1 	ASAVDG-KSDPPAATVEAAKASLSLDDKRTLLKKVLRSPQFNQALASLTMAIRDGGLPSI
AN1167.1  	EDQLAS-QQRPETA--QEVLDALNLSQKRNILRKILRSPQFTQSLASLTVAIRDGGLPSI
          	 .   . :  * :*    .  ::.  :*: :*:::****** *:*:**::*:******.*

MG04443.1 	AEALGVKVENGGLVRGGSVPLGGGEAVEAFVEGVKKEALDKKR-------
NCU02634.1	SAALGIQVENGGMVRGGMVPLGGGDAVEAFVEGVKKTVQKK---------
FG01009.1 	ADALSVKVQDGGYLRGSGMPLGGGQAVEAFVNGVKKTVEEEKK-------
AN1167.1  	SEALKIPVENGGFMRRGGVPLGGGDAMKAFIQGVRDYVKDSTQGNRMETD
          	: ** : *::** :* . :*****:*::**::**:. . ... ..  .:.
```
